# Trans-arterial and trans-venous interventional radiology for an elderly patient with life-threatening pelvic injury after accidental falling due to life-threatening cardiac arrhythmia: a case report

**DOI:** 10.4076/1757-1626-2-6222

**Published:** 2009-07-20

**Authors:** Junya Morozumi, Takao Arai, Shöichi Ohta

**Affiliations:** Department of Emergency and Critical Care Medicine, Tokyo Medical University Hospital Hachioji Medical CenterTokyo, 193-0998Japan

## Abstract

**Introduction:**

A 79-year man, admitted to our emergency department after accidental falling with syncope, had pelvic fractures and complete atrioventricular (AV) block.

**Case presentation:**

He received transvenous pacer placement for complete AV block and required hemodynamic stability. His heart rate was successfully controlled. However, secondary deterioration of his hemodynamics concerning of pelvic fractures was occurred and immediate trancatheter arterial embolization was performed. Final angiography showed no findings of bleeding and he was discharged intensive care unit in good condition.

**Conclusion:**

Combined transarterial and transvenous interventional radiology is an effective and safety resuscitation technique for an elderly with secondary life-threatening injury after accidental falling due to life-threatening cardiac arrhythmia.

## Introduction

Syncope is a clinical symptom which is characterized by a transient loss of consciousness, usually leading to accidental falling about one third of the population aged 65 or more [1,2]. Of 5-10% of the population result in secondary severe injuries after accidental falling, which is recognized as one of the lethal complications of transient loss of consciousness [3,4]. In the elderly, the number of patients who fall due to syncope has also increased [5,6] and more than 90% of hip fractures are the result of a fall [7].

In the present study, we treated an elderly patient with a life-threatening pelvic injury after accidental falling secondary to syncope due to complete atrioventricular (AV) block using interventional radiology (IVR).

## Case presentation

**Figure 1.**
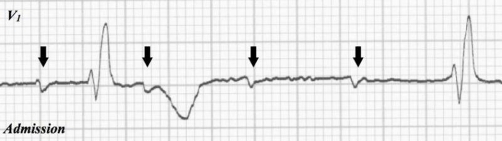
Electrocardiogram of the patient showing complete atrioventricular block. P wave (black arrow).

**Figure 2.**
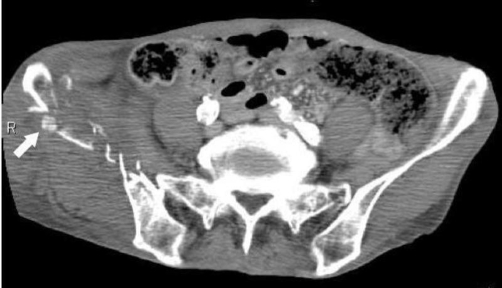
Computed tomography showed pelvic fractures with contrast medium extravasation (white arrow).

A 79-year-old Asian man was admitted to our emergency department (ED) after accidental falling with syncope from 2nd floor to the ground at his house. On examination, he had an E3V3M5 Glasgow Coma Scale score with restlessness, a presenting blood pressure of 104/55 mmHg, heart rate of 35 beats/minute, and a respiratory rate of 24/minute on admission. He immediately received initial trauma resuscitation on the basis of the Advanced Trauma Life Support guidelines [8]. Initial electrocardiogram showed complete AV block in the ED (Figure 1). Though he underwent administration of 0.5 mg of atropine and transcutaneous pacing immediately in primary care, his heart rate showed no improvement. Focused assessment with sonography for trauma and chest X-ray in the ED were interpreted as normal. Pelvic X-ray showed pelvic fractures in the sacrum, right pubic rami, and the ileum. Contrast-enhanced computed tomography (CT) scan of the chest, abdomen and pelvis showed hematoma on the right side of the pelvic cavity that compressed the bladder in the obturator internus, the iliacus and in the gluteus medius muscle of the fracture area and extrapelvic hematoma with contrast medium extravasation (Figure 2). The dislocation of the right elbow was also determined. The injury severity score, which is based on the abbreviated injury scale (AIS) severity values and derives from the sum of the squares of the highest AIS values from each of the 3 most severely injured body regions, was 20.

**Figure 3.**
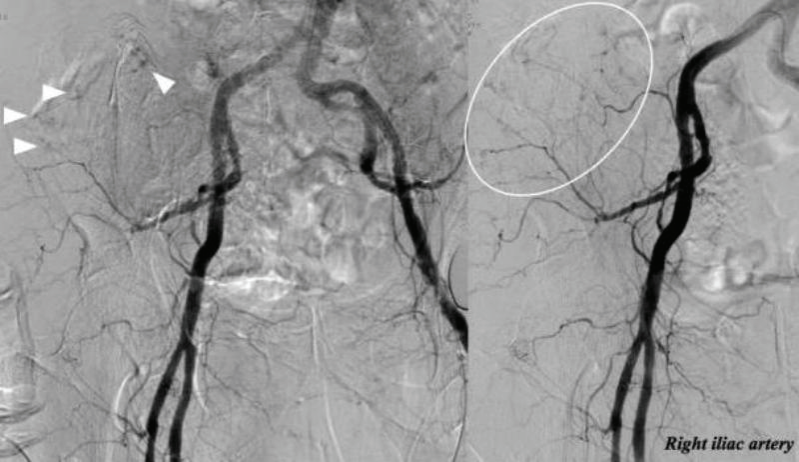
Pelvic angiography showed multiple contrast medium extravasation (white arrow and white circle).

**Figure 4.**
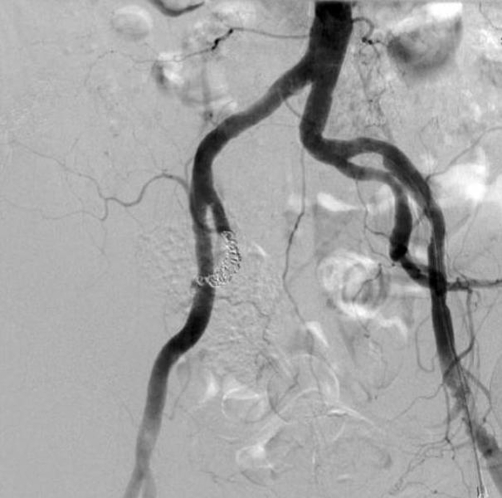
After completion of transcatheter arterial embolization, the contrast medium extravasation halted.

Despite administration of initial fluid resuscitation using Ringer’s lactate, his systolic blood pressure (SBP) deteriorated to 70 mmHg and heart rate of 30 beats/minutes. We were concerned about cardiogenic shock or hemorrhagic shock, and therefore decided to place a transvenous pacer for complete AV block and to perform transcatheter arterial embolization (TAE) for the pelvic injury with massive fluid resuscitation in primary trauma resuscitation. We initially transferred him to the angiography room and initiated IVR to place the transvenous pacer because we found no improvement of his heart rate in spite of administration of atropine and placement of a transcutaneous pacemaker in the ED. The transvenous pacer was successfully placed for 1 hour, and his SBP had recovered to 100 mmHg. After completion of placement of the transvenous pacer, his heart rate was controlled at 60 beats/minute. However, his hemodynamic status soon deteriorated to a SBP of 79 mmHg. We continuously initiated TAE for secondary deterioration of his hemodynamic status concerning of pelvic injury. Initial pelvic angiography showed multiple contrast medium extravasation from the branches of the right internal iliac artery (Figure 3). On completion of TAE, using stainless coils and gelatin sponge particles for the pelvic fractures, angiography showed no findings of bleeding (Figure 4). The patient required hemodynamic stability with a SBP of 150 mmHg and heart rate was controlled at 60 beat/minutes. After completion of TAE, he was admitted to our intensive care unit (ICU). At 12 hours after admission, he received a transfusion of 5 units of red blood cells and 10 units of fresh frozen plasma and his hemodynamic status remained stable. He was discharged ICU on hospital day 2, and his blood culture from admission to 24 hours after admission was detailed in Table 1.

**Table 1.** Patient’s clinical data during primary trauma resuscitation

Variables (normal)	Pre IVR	Post IVR	At 12 hours after admission	At 24 hours after admission
Red blood cells (430-550 × 10^4^/μl)	221	347	367	368
Hemoglobin (13.5-175.5 g/dl)	7.4	11.3	11.8	12.0
Hematocrit (39-52%)	22	32	34	34
PLATE (15-35 × 10^4^/μl)	15.7	15	12.5	11.7
PT (sec)	14.9	14.3	14.0	14.1
PT count (sec)	12.6	12.6	12.6	12.6
APTT (sec)	42.1	43.2	43.3	42.5
APTT count (sec)	35.3	35.3	37.9	37.9
pH	7.319	7.454	7.412	7.528
Basic deficit (mmol/l)	−2.4	1.9	0.9	0.5

IVR, interventional radiology; PT, prothrombin time; APTT, activated partial thromboplastin time.

## Discussion

This patient had potentially fatal complications after accidental falling due to complete AV block, suffering severe pelvic injury, resulting in secondary hemorrhagic shock. Syncope is a clinical symptom, which is characterized by a transient, and self-limited loss of consciousness due to temporary reduction of cerebral perfusion [2]. For the elderly, syncope is a common problem in the health care setting, and attacks account for at least 6% of hospital visits [9]. Moreover, syncope usually leads to a secondary accidental fall, and the risk of accidental falling has been found to be greater than 20% per year for the elderly over 65 years [10]. In most cases, the syncopal condition is benign but in a minority, the condition can be sometimes life-threatening. Bartoletti et al. reported that 33% of the prevalence of secondary trauma among the elderly patients with syncope is due to cardiac arrhythmias [11]. According to the European Society of Cardiology Guidelines on Syncope, complete AV block was one of the life-threatening cardiac arrhythmia causes of syncope and the most common reason for pacemaker implantation [12]. In the present case, he soon received administration of atropine and placement of a transcutaneous pacemaker for complete AV block on the basis of the 2005 American Heart Association guidelines [13]. However, no hemodynamic improvement was recognized. Thus, we decided to place a transvenous pacer using IVR instead of surgical pacemaker implantation due to the hemodynamic instability in this elderly patient.

However, a hematoma with contrast medium extravasation by pelvic fractures was detected on CT scan, which meant a life-threatening situation requiring surgical or radiological intervention. Control of the arterial and venous hemorrhage, and prevention of exacerbation of his hemodynamic status and coagulation state was deemed essential. For pelvic injuries, optimal therapy in the face of bleeding pelvic injury is still controversial. The options for external pelvic fixation using a pelvic C clamp or an external fixator, which is called damage control orthopedics, may be considered for definitive treatment of pelvic fracture, in Europe [14]. These external pelvic interventions can effectively provide a temporary solution of the problem of hemorrhage of venous origin. However, delay in TAE for external pelvic fixation in patients with pelvic fractures would have delayed embolization in the face of ongoing arterial bleeding [15]. Reports that damage control orthopedics for multiple trauma, especially including head trauma, increased pulmonary complications, acute respiratory distress syndrome, or deterioration of outcome for multiple trauma patients have increased [16]. These complications or exacerbation of anemia due to arterial hemorrhage might increase the risk of the exacerbation of cardiac arrhythmia and cardiac function.

TAE appears to achieve its effect by controlling the arterial hemorrhage and allowing the tamponade effect of the hematoma to control venous hemorrhage [17]. Moreover, patients who undergo TAE for pelvic injury tend to be older. Kimbrell et al. suggested that an age of 60 years or older has a high likelihood of active retroperitoneal hemorrhage, regardless of the presumed hemodynamic stability on angiography, and the elderly over 60 years was the only independent predictor of the need for TAE for pelvic injury [18]. They also suggested that TAE is highly effective and less invasive in controlling hemorrhage in cases of pelvic injuries, which are difficult to manage by surgical intervention.

In the present case, surgical intervention was difficult due to his hemodynamic status. Therefore, it necessary to perform transvenous IVR for life-threatening cardiac arrhythmia immediately, and simultaneously to diagnose of arterial hemorrhage and perform TAE, if arterial hemorrhage was detected, in order to prevent metabolic and heart failure due to persistent hemorrhage were the optimal treatment options. We strongly emphasize that TAE is an effective and safe resuscitation technique for the elderly patient with secondary pelvic fractures after accidental falling due to life-threatening cardiac arrhythmia which needs interventional treatment.

## Abbreviations

AIS: abbreviated injury scale
AV: atrioventricular
CT: computed tomography
ED: emergency department
ICU: intensive care unit
IVR: interventional radiology
SBP: systolic blood pressure
TAE: transcatheter arterial embolization
